# Coronary Artery Bypass Grafting Is Rarely Done in the Acute Care of ST-elevation Myocardial Infarction Patients Treated by Emergency Medical Services

**DOI:** 10.5811/westjem.35271

**Published:** 2025-05-20

**Authors:** Jake Toy, Caroline Lauer, Amy H. Kaji, Joseph L. Thomas, Nichelle Megowan, Nichole Bosson, Marianne Gausche-Hill, Puneet Dhawan, Robert A. Kloner, Sara Rasnake, William French, Shira Schlesinger

**Affiliations:** * Los Angeles Emergency Medical Services Agency, Santa Fe Springs, California; † Harbor-UCLA Medical Center, Department of Emergency Medicine, Torrance, California; ‡ Harbor-UCLA Medical Center, The Lundquist Institute for Biomedical Innovation at Harbor-UCLA, Torrance, California; § David Geffen School of Medicine at UCLA, Los Angeles, California; ¶ Huntington Medical Research Institutes, Pasadena, California; || Harbor-UCLA Medical Center, Department of Cardiology, Torrance, California; # Harbor-UCLA Medical Center, Department of Cardiothoracic Surgery, Torrance, California; ** Keck School of Medicine of University of Southern California, Los Angeles, California

## Abstract

**Introduction:**

The use of coronary artery bypass grafting (CABG) for primary revascularization during the acute care of ST-elevation myocardial infarction (STEMI) patients has declined significantly in the past decade; but there is little data to determine whether there has been a change in the use of CABG for STEMI patients treated by emergency medical services (EMS). In this study we described the incidence of urgent or emergent CABG for STEMI patients treated in a large, regionalized cardiac care system.

**Methods:**

We obtained data obtained for patients transported by EMS between January 2011–December 2022 who were diagnosed with acute STEMI on prehospital or emergency department (ED) electrocardiogram and taken for primary diagnostic catheterization. All STEMI patients were transported by EMS to one of 34 STEMI receiving centers (SRC) in a regionalized cardiac care system, all of which are required to maintain onsite cardiac surgery as a condition of their SRC designation. Patients were considered to have undergone urgent or emergent CABG if it was performed within 72 hours of the primary diagnostic cardiac catheterization. We excluded patients if no diagnostic catheterization was performed or if CABG was performed >72 hours after diagnostic catheterization. The primary outcome was the incidence of urgent or emergent CABG. Patients were further stratified by time between diagnostic catheterization and CABG (<24 hours, 24–48 hours, 48–72 hours).

**Results:**

A total of 28,349 patients were transported by EMS and diagnosed with an acute STEMI during the study period. Only 384 (1.35%) patients underwent CABG within 72 hours of diagnostic catheterization: 268 (0.95%) underwent CABG in <24 hours; 71 (0.25%) in 24–48 hours, and 45 (0.16%) in 48–72 hours. The median age of patients undergoing CABG was 64 years (interquartile range 58–72). Twenty-eight (7.3%) experienced prehospital cardiac arrest, and eight (2.1%) required vasopressors. Prior to undergoing CABG, 137 patients (36%) underwent primary percutaneous coronary intervention. The proportion of patients undergoing CABG within 72 hours remained relatively stable between 2011–2022 at 1.19% and 1.96%, respectively.

**Conclusion:**

Urgent or emergent CABG remained infrequently performed for acute STEMI patients after primary diagnostic catheterization. There was little change in the percentage of STEMI patients who received CABG within 72 hours of diagnostic catheterization over the past decade. These findings suggest that regional or local policies requiring on-site cardiac surgery at SRCs may be reconsidered.

## INTRODUCTION

The use of emergency coronary artery bypass grafting (CABG) for revascularization during the acute phase of ST-elevation myocardial infarction (STEMI) care has decreased over the past decade.^1^,^2^ Primary percutaneous coronary intervention (PCI) is the modality of choice for myocardial reperfusion in STEMI patients; emergency CABG is typically necessitated as a rescue modality for patients with ongoing ischemia, shock, coronary anatomy not amenable to PCI, post-MI mechanical complications, or PCI complications.^3^,^4^ In the United States, use of emergency CABG for primary revascularization after STEMI care has declined to less than 5% annually.^1^,^2^,^5^,^6^

Historically, all acute STEMI patients treated by emergency medical services (EMS) were transported to receive primary PCI at hospitals with on-site cardiac surgery backup.^7^ Nonetheless, more recent evidence suggests comparable patient outcomes after primary PCI at STEMI receiving centers (SRC) with and without on-site cardiac surgery.^8^ From these studies, it was found that there were no differences observed in the rates of emergency cardiac surgery or mortality at SRCs without cardiac surgery backup.^8^ National policy guidelines by the Society for Cardiovascular Angiography and Interventions, American College of Cardiology Foundation, and American Heart Association (AHA) have aligned with this evidence and now support the use of primary PCI for STEMI patients at SRCs without on-site cardiac surgery if proper transfer agreements to facilities with cardiac surgery have been established.^9^,^10^ Despite this change in national recommendations, policies for SRC designations remain variable between states and in major metropolitan centers in California.^11^–^13^ Furthermore, it is increasingly challenging to transfer patients rapidly between hospitals for those patients needing emergent specialty intervention. Overall, delays in care can lead to worse outcomes.^14^ This has implications for both hospital services and EMS routing policies within regional cardiac systems of care.

The purpose of this study was to quantify the current incidence of urgent or emergent CABG performed for EMS-treated patients diagnosed with an acute STEMI and taken for primary diagnostic catheterization in a large regionalized cardiac care system over the past decade. We further sought to assess times from primary diagnostic catheterization to urgent or emergent CABG to understand whether this group of patients could be safely transferred for higher level of care within a regionalized system. These study results may guide the designation of SRCs within a regionalized cardiac care system and are relevant to administrators and physicians from the fields of EMS, emergency medicine, and cardiology.

## METHODS

### Study Design

This retrospective study quantified the incidence of urgent and emergent CABG among EMS-transported patients who were diagnosed with an acute STEMI in the ED and taken for primary diagnostic catheterization within the regionalized cardiac care system in Los Angeles County. We followed the relevant retrospective chart review guidelines set forth by Worster el al and established a case selection criteria, defined variables, described the medical database, and outlined the sampling method used.^15^ This study was determined to be exempt by the Institutional Review Board at the Lundquist Institute for Biomedical Innovation at Harbor-UCLA Medical Center.

**Population Health Research Capsule**

What do we already know about this issue?

*The incidence of cardiac artery bypass grafting (CABG) during acute STEMI care is rare, yet policies continue to require 24/7 cardiac surgery coverage at STEMI centers.*

What was the research question?

*What is the incidence of CABG at <72 hours for STEMI patients?*

What was the major finding of the study?

*Annually, <2% of EMS-treated STEMI patients received urgent or emergent CABG at <72 hours after primary diagnostic catheterization.*

How does this improve population health?

*Designation of STEMI receiving centers without requiring 24/7 cardiac surgery coverage may increase access to STEMI care.*

### Population and Setting

Los Angeles County has a population of nearly 10 million in a region spanning over 4,050 square miles and including rural, suburban, and urban areas.^16^ The EMS 9-1-1 response is provided by 29 fire departments and one law enforcement agency, with Basic and Advanced Life Support units.^17^ The Los Angeles County EMS Agency provides medical oversight and standardized treatment protocols used by all EMS responders operating within the county. The county uses a combination of direct and indirect medical oversight, with 21 base hospitals providing direct oversight for patients with high-risk or complex presentations.

The Los Angeles County EMS Agency oversees our regionalized cardiac care system; paramedics transport all patients with STEMI on prehospital electrocardiogram (ECG) to one of 34 designated SRCs.^18^ Per current county treatment protocols, ECGs with STEMI detected by the evaluating paramedic or by automated report of the EMS monitor-defibrillator are transmitted to the nearest designated SRC for physician review and potential activation of the cardiology catheterization team. The EMS agency designates all SRCs in Los Angeles County and requires each SRC to have cardiac catheterization and cardiothoracic surgery services 24 hours/7 days a week/365 days a year. All SRCs must also submit data to the Los Angeles County EMS Agency for system quality improvement for patients transported by EMS to a SRC with suspected acute STEMI or a confirmed STEMI in the ED within one hour of arrival.

### Patient Population

For this study, we included adult patients who were transported by EMS between January 1, 2011–December 31, 2022 to a designated SRC, and who were diagnosed with an acute STEMI by the cardiologist at the SRC based on prehospital or ED ECG and taken for primary diagnostic cardiac catheterization. Patients were excluded if they were <18 years of age, did not receive a diagnostic catheterization prior to CABG during their index hospitalization (either due to false-positive prehospital STEMI activation based on the prehospital ECG, contraindication to catheterization, or refusal), or if the CABG date and time were missing.

### Data Collection

We etracted data from consecutive EMS-transported acute STEMI patients from the county SRC database, which combines prehospital information from electronic patient care reports (eg, field care data) and from hospital electronic health records (eg, hospital outcome data). We extracted demographic variables including age and sex, and clinical variables including whether the patient experienced an out-of-hospital cardiac arrest, and whether the patient received vasopressors prior to PCI or CABG, primary diagnostic catheterization, PCI, and CABG during hospitalization. We also extracted the date and time of primary diagnostic catheterization, PCI, CABG, hospital disposition, hospital length of stay, and survival to hospital discharge. The interval between primary diagnostic catheterization and CABG was measured in minutes. Reasons for not performing primary PCI as reported by the interventional cardiologist were also extracted.

For patients transported between July 2019–December 2022, changes in the structure of the regional SRC database allowed for an additional variable to be extracted from this time period, which was the urgency of the CABG (ie, salvage, emergent, urgent, elective).^19^ This variable is defined as follows: salvage refers to the presence of ongoing cardiopulmonary resuscitation enroute to the operating room or prior to induction of anesthesia; emergent, any procedure required for a patient with ischemic or mechanical dysfunction who was not responsive to any therapy except surgery; urgent, any surgery required during the same hospitalization to minimize patient deterioration; and elective, surgery conducted on a patient whose cardiac function has been stable prior to the operation. Level of urgency was based on the judgment of the primary cardiothoracic surgeon.

### Outcomes

The primary outcome was the proportion of EMS-transported patients who underwent CABG within 72 hours after an acute STEMI diagnosis in the ED and primary diagnostic catheterization. The secondary outcome was the proportion who underwent CABG based on stratified time intervals (<24 hours, 24–48 hours, and 48–72 hours). We considered CABG performed within 72 hours of primary diagnostic catheterization to be urgent or emergent. Review of prior literature found no temporal definition of urgent or emergent CABG that could be easily applied to our dataset.^20^ Generally, an emergency CABG during the acute phase of STEMI care has been defined as an unscheduled surgical procedure performed due to ongoing ischemia and/or refractory cardiogenic shock not amenable to PCI.^4^,^21^ While no specific time cut-off has been suggested, one study defined emergency CABG as any operation that was performed before the next working day.^22^ We chose a 72-hour cut off to capture those patients who underwent an urgent or emergent CABG early in their hospitalization. When creating time interval stratifications, we chose the <24-hour interval to represent a period during which transfer of a critically ill cardiac patient to higher level of care would be challenging within our system.

### Analysis

We reported continuous variables as medians and interquartile ranges (IQR) and categorical variables as frequencies and percentages. For continuous variables, we used the Kruskal-Wallis rank-sum test as the test for statistical significance; and for categorical variables, we used the chi-squared test or Fisher exact tested. *P*-values <0.05 were considered statistically significant. We conducted all analyses and data visualization via R software v 4.3.3 (RStudio, Posit, PBC, Boston, MA).

## RESULTS

**Figure 1:**
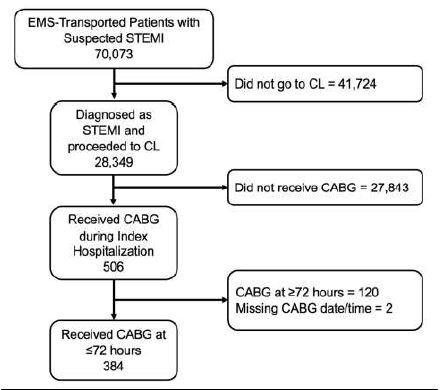
Flow diagram. *EMS*, emergency medical services; *STEMI*, ST-elevation myocardial infarction; *CABG*, coronary artery bypass graft; *CL*, catheterization lab.

Of 70,073 EMS-transported patients with a STEMI-activation based on the prehospital or ED ECG during the 12-year study period, 28,349 were diagnosed with an acute STEMI in the ED by the cardiologist and were taken for primary diagnostic cardiac catheterization. Of these 28,349 patients transported by EMS with confirmed STEMI, who represent the study population, 506 (0.82%) patients underwent urgent or emergent CABG during their hospital course. After exclusion of patients receiving CABG outside the specified 72-hour interval and those with incomplete data (Figure 1), 384 (1.35%) patients received CABG within 72 hours of primary diagnostic catheterization; 268 (0.95%) received CABG within 24 hours, 71 (0.25%) in 24–48 hours, and 45 (0.16%) in 48–72 hours. Less than half of patients received PCI prior to CABG in the <24-hour and 24–48-hour subgroups, and in slightly more than half in the 48–72-hour subgroup. Demographic and clinical characteristics for the study population are shown in Table 1.

The median intervals from primary diagnostic catheterization to CABG are reported in Table 2. For patients in the <24-hour group, the median interval to CABG was 303 minutes (5.1 hours). The most common reported reasons for not performing PCI prior to CABG was that the patient required CABG for multivessel disease, frequently with the use of an intra-aortic balloon pump (Table 3).

**Figure 2:**
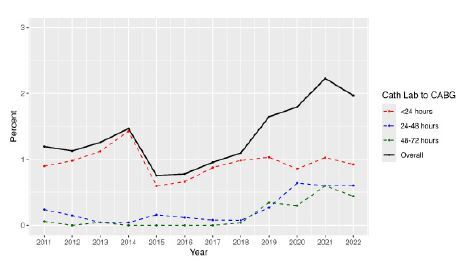
ST-elevation myocardial infarction patients transported by emergency medical services and to CABG after primary diagnostic catheterization lab. Patients are stratified by the time interval (<24 hours, 24–48 hours, and 48–72 hours) *EMS*, emergency medical services; *STEMI*, ST-elevation myocardial infarction; *CABG*, coronary artery bypass graft; *Cath Lab*, catheterization lab.

**Figure 3:**
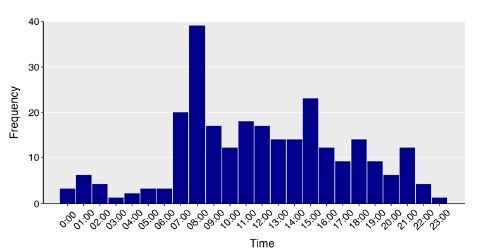
Time of day when the coronary artery bypass graft operation took place for the <24-hour group.

When evaluating temporal trends between 2011–2022 (Figure 2), the overall proportion of EMS-transported patients diagnosed with an acute STEMI that underwent CABG within 72 hours remained relatively stable between 2011–2022 at 1.19% and 1.96%, respectively, and this finding was similar in the other time intervals assessed. When assessing the time of day that CABG took place, 204 (8%) took place between the hours of 7 am and 6 pm (Figure 3).

Among EMS-transported patients diagnosed with an acute STEMI between July 2019–December 2022 who underwent CABG, a pre-planned subanalysis to understand perceived CABG urgency demonstrated that the majority of cases in all time interval subsets were classified as “urgent.” Of those categorized as “emergent,” 90% received CABG within 24 hours. A complete breakdown is shown in Table 4.

## DISCUSSION

In our investigation of EMS-transported patients who were diagnosed with an acute STEMI in the ED and taken for primary diagnostic catheterization we found that emergency CABG was infrequent. Over the 12-year study period, the proportion of acute STEMI patients undergoing urgent or emergent CABG was consistently below 3% each year. Among patients undergoing operative intervention within 24 hours, few required vasopressor support prior to CABG, and the median time to CABG was approximately five hours. These results suggest that the potential rate of emergent interfacility transfers for higher level of care for urgent or emergent CABG would be low in our system and aligns with prior evidence suggesting that primary PCI may be performed at hospitals without cardiac surgery backup if appropriate transfer agreements are in place.^8^

In the United States, the overall incidence of emergency CABG during the acute phase of STEMI care has been evaluated in multiple prior studies. A nationwide study by Keeling and colleagues reported that the incidence of emergent CABG for all STEMI patients remained consistently below 5% between 2005–2017 in the US.^2^ Other studies have reported similarly low rates of CABG for all STEMI patients in the US.^1^,^5^,^6^ This study focused specifically on EMS-transported patients who were diagnosed with an acute STEMI in the ED by the treating cardiologist and taken for primary diagnostic catheterization, and also found consistently low rates of urgent or emergent CABG.

Multiple prior studies have evaluated outcomes after primary PCI for STEMI patients at centers with and without on-site cardiac surgery.^8^,^23^,^24^ One prior randomized trial in 2004 found that mortality outcomes were similar for MI patients who received primary PCI at a hospital without on-site cardiac surgery compared to those transferred for primary PCI.^24^ Additionally, a 2015 meta-analysis by Lee and colleagues compared outcomes of primary PCI after STEMI at hospitals with and without on-site cardiac surgery using 23 studies in an analysis totaling >1 million patients.^8^ The authors found similar mortality outcomes in patients undergoing primary PCI for STEMI between hospitals with and without on-site cardiac surgery, and that the need for emergency cardiac surgery was rare in a pooled analysis (2.4% and 1.5% in hospitals with and without cardiac surgery, respectively). These studies provide evidence that primary PCI at hospitals without on-site cardiac surgery is likely safe and feasible in the modern era.

In 2021, the AHA released policy guidelines for the implementation of STEMI systems of care and outlined new recommended levels of care.^10^ These guidelines described that primary heart attack centers (PHAC) must have 24/7/365 PCI capability and cardiac surgery backup is not required, while comprehensive heart attack centers (CHAC) must have 24/7/365 PCI capability and on-site cardiac surgery. The PHACs must have transfer agreements with CHACs to facilitate timely advanced cardiac care when needed. Our results demonstrated consistently low numbers of patients undergoing urgent or emergent CABG after a diagnosis of acute STEMI and primary diagnostic catheterization; the results from this study support the guidance and levels of care outlined in these national recommendations.

Nonetheless, widespread implementation of these guidelines in regional EMS systems has been slow. California state regulations allow SRCs to perform PCI without on-site cardiac surgery if written transfer agreements are in place.^25^ Within each county in the state of California, local EMS agency policy governs EMS operations; thus, the practice of EMS clinicians may vary between regions. The LA County EMS Agency policy requires all SRCs to have on-site cardiac surgery. Among the 34 local EMS agencies (LEMSA) in California, 21 allow SRCs to perform primary PCI without on-site cardiac surgery, seven require on-site cardiac surgery at all SRCs, and three allow SRCs to perform primary PCI without on-site cardiac surgery only with permission from the LEMSA (Supplement). The reasons for this variation in policy are likely due to region-specific considerations (ie, rurality, population density, number of hospitals, interfacility transfer policies, and resources), a lag effect between national recommendations and policy implementation, a desire to adhere to the highest level of care recommendations set by the AHA (ie, CHACs), and existing community standards and expectations.^26^

The ability to rapidly and safely transfer an acute STEMI patient requiring urgent or emergent CABG from an SRC without on-site cardiac surgery to a higher level of care has been a persistent concern. Emergency medical services systems continue to experience ambulance shortages leading to long wait times for ambulances, including critical care transports.^27^,^28^ These concerns have likely contributed to hesitancy surrounding the removal of the requirement for on-site cardiac surgery at SRCs in some areas. In our study, the 24-hour time frame is an interval during which we anticipated it would be logistically challenging to arrange critical care transport and prepare the patient for surgery at the receiving facility. Only 23 (0.92%) EMS-transported STEMI patients underwent CABG in the first 24 hours of hospitalization across the SRCs during 2022, equating to fewer than two patients per month across a system of 34 SRCs. These patients rarely required vasopressor support prior to CABG. Further, our subanalyses found no cases of CABG marked with an urgency of “salvage” and a majority of cases with CABG within 24 hours had operative case start times during daytime hours. These findings build upon prior investigations in the US^1^,^2^,^5^ and support wider acceptance of SRCs without on-site cardiac surgery back up as along as appropriate transfer aggrements are in place.

## LIMITATIONS

This study was not free from limitations. We were unable to stratify our analysis by patients needing urgent or emergent CABG for primary revascularization vs PCI complications due to limitations in the available data. Nonetheless, only 0.4% (136/28,349) of STEMI patients during the entire study period underwent CABG after PCI and, thus, the rate of PCI complication leading to CABG could be, at most, 0.4% in our system. In our subanalysis between July 2019–December 2022, we also noted the inclusion of three cases deemed elective by the treating cardiothoracic surgeon. Given that we did not have data on reported urgency for the entire study population, we were unable to exclude these rare elective CABG cases performed within 72 hours of prior diagnostic catheterization.

Additionally, SRCs in our EMS system had on-site cardiac surgery; therefore, actual emergent interfacility transfer rates could not be measured. However, potential emergent interfacility transfer rates for a higher level of care may be extrapolated from our results. Finally, we evaluated only the incidence of CABG in patients with confirmed STEMI diagnosed in the ED, rather than the larger population of patients transported to STEMI receiving centers with prehospital-suspected STEMI. Including this broader population would have further decreased the proportion of prehospital patients at risk for requiring urgent or emergent cardiac surgery during their index visit.

## CONCLUSION

Among EMS-treated patients who were diagnosed with an acute STEMI and taken for primary diagnostic catheterization, the incidence of urgent or emergent cardiar artery bypass graft within 72 hours of primary diagnostic catheterization was low and remained low during the 12-year study period. Our results showed that on average there was less than one case of emergent CABG performed at each facility annually. Based on these findings, regional or local policies requiring onsite cardiac surgery at STEMI receiving centers may be reconsidered.

## Supplementary Information



## Figures and Tables

**Table 1:** Descriptive characteristics.

	Overall N = 384 n (%)	<24 hours n = 268 n (%)	24–48 hours n = 71 n (%)	48–72 hours n = 45 n (%)	*P*-value
Age (years), median (IQR)	64 (58, 72)	63 (57, 72)	65 (60, 73)	65 (58, 72)	0.46
Male	314 (82)	219 (82)	55 (77)	40 (89)	0.30
Cardiac arrest	28 (7.3)	20 (7.5)	5 (7.0)	3 (6.7)	>0.99
Received vasopressors	8 (2.1)	6 (2.2)	2 (2.8)	0 (0)	0.63
Received PCI	137 (36)	89 (33)	25 (35)	23 (51)	0.07
Survived to hospital discharge	348 (91)	236 (88)	67 (96)	45 (100)	0.007
Hospital length of stay (days), median (IQR)	7.0 (5.0, 10.0)	7.0 (5.0, 9.0)	7.0 (6.0, 10.0)	8.0 (7.0, 10.0)	<0.001

Note: There was one missing value for sex, and two missing values for survival to hospital discharge and hospital length of stay.

*IQR*, interquartile range; *PCI*, percutaneous coronary intervention.

**Table 2:** Average time from primary diagnostic catheterization to coronary artery bypass graft.

	Overall N = 384 Median (IQR)	<24 hours n = 268 Median (IQR)	24–48 hours n = 71 Median (IQR)	48–72 hours n = 45 Median (IQR)
Cath Lab to CABG (hours)	13.58 (3.42, 28.28)	5.05 (2.67, 14.43)	34.12 (28.02, 41.03)	62.03 (55.65, 66.8)

*IQR*, interquartile range; *CABG*, coronary artery bypass graft; *cath lab*, cardiac catheterization laboratory.

**Table 3:** Reason for not performing percutaneous coronary intervention prior to coronary artery bypass graft.

	Overall N = 247 n (%)	<24 hours n = 179 n (%)	24–48 hours n = 46 n (%)	48–72 hours n = 22 n (%)
Reason PCI was not performed				
Aortic dissection	1 (0.4)	1 (0.6)	0 (0)	0 (0)
Candidate for CABG/multivessel disease	229 (93)	162 (91)	46 (100)	21 (95)
Coronary artery dissection	1 (0.4)	1 (0.6)	0 (0)	0 (0)
Difficult cath/unable to cannulate/dilate vessel/cross lesion/locate artery	13 (5.3)	12 (6.7)	0 (0)	1 (4.5)
No reason provided	1 (0.4)	1 (0.6)	0 (0)	0 (0)
Other	2 (0.8)	2 (1.1)	0 (0)	0 (0)

*PCI*, percutaneous coronary intervention; *CABG*, coronary artery bypass graft; *cath*, catheterization.

**Table 4:** A subanalysis for reported urgency of coronary artery bypass graft operation.

	Overall N = 178 n (%)	<24 hours n = 83 n (%)	24–48 hours n = 51 n (%)	48–72 hours n = 44 n (%)
CABG Status				
Urgent	135 (76)	46 (56)	46 (90)	43 (98)
Emergent	40 (22)	36 (43)	4 (8)	0 (0)
Elective	3 (2)	1 (1)	1 (2)	1 (2)

Note: Data was only present for July 2019–December 2022. Additionally, there were zero cases reported as “salvage” during this period.

*CABG*, coronary artery bypass graft.
