# Supplementary material for: Coronary Artery Bypass Grafting Is Rarely Done in the Acute Care of ST-elevation Myocardial Infarction Patients Treated by Emergency Medical Services
Source: West J Emerg Med. 2025 May 20;26(3):729–36. doi: 10.5811/westjem.35271 (PMC12208032; doi:10.5811/westjem.35271)
Supplement: Supplementary file 1 [file wjem-26-729-s001.docx]

**Supplemental Material A.** STEMI receiving center designation criteria in California.

|  | | |
| --- | --- | --- |
| **Agency** | **Require On-site Cardiac Surgery** |  |
| ***State*** |  |  |
| California Code of Regulations^1^ | No |  |
| ***County (Local EMS agencies)*** |  |  |
| Alameda County EMS^2^ | No |  |
| Central California EMS Agency^3^ | No |  |
| Coastal Valleys EMS Agency^4,5^ | Yes |  |
| Contra Costa Health Services^6^ | Yes (may be waived) |  |
| El Dorado County EMS Agency^7^ | Yes (may be waived) |  |
| Imperial County EMS Agency^8,9^ | No |  |
| Inland Counties Emergency Medical Agency^10^ | Yes |  |
| Kern County Public Health^11^ | No |  |
| Los Angeles County Health Services^12^ | Yes |  |
| Marin County EMS Agency^13^ | No |  |
| Merced County Department of Public Health^14^ | Yes |  |
| Monterey County EMS Agency^15^ | No |  |
| Mountain Valley EMS Agency^16^ | No |  |
| Napa County California^17,18^ | No |  |
| North Coast EMS^19^ | No |  |
| Northern California EMS Inc.^20,21^ | No |  |
| Orange County Health Care Agency^22^ | Yes |  |
| Riverside County EMS Agency^23^ | Yes (may be waived) |  |
| Sacramento County EMS^24^ | No |  |
| San Benito EMS^25^ | No |  |
| San Diego County EMS Office^26^ | No |  |
| San Francisco EMS Agency^27^ | Yes |  |
| San Joaquin EMS Agency^28^ | Yes |  |
| San Luis Obispo EMS Agency^29^ | No |  |
| San Mateo County EMS^30^ | No |  |
| County of Santa Barbara EMS Agency^31^ | No |  |
| County of Santa Clara EMS^32^ | No |  |
| County of Santa Cruz Health Services Agency^33^ | No |  |
| Sierra-Sacramento Valley EMS^34^ | No |  |
| Solano County EMS^35^ | No |  |
| Stanislaus County EMS Agency^36^ | No |  |
| Tuolumne County EMS^37^ | Yes |  |
| Ventura County EMS^38^ | No |  |
| Yolo County EMS^39^ | No |  |

Note: Policies were reviewed by two reviewers in February 2024.

*EMS*, emergency medical services, *SRC*, ST-elevation myocardial infarction receiving center.
